# Depressed Kallikrein Generation in Women With Preeclampsia: A Matched Cross-Sectional Study

**DOI:** 10.3389/fmed.2022.896811

**Published:** 2022-06-06

**Authors:** Anne Cathrine Godtfredsen, Jørgen Brodersen Gram, Stephanie Thuy Duong Pham, Britta Blume Dolleris, Jan Stener Jørgensen, Johannes Jakobsen Sidelmann, Yaseelan Palarasah

**Affiliations:** ^1^Department of Gynecology and Obstetrics, University Hospital of Southern Denmark, Esbjerg, Denmark; ^2^Unit for Thrombosis Research, Department of Regional Health Research, University of Southern Denmark, Esbjerg, Denmark; ^3^Department of Clinical Biochemistry, University Hospital of Southern Denmark, Esbjerg, Denmark; ^4^Department of Cancer and Inflammation Research, University of Southern Denmark, Odense, Denmark; ^5^Department of Gynecology and Obstetrics, University Hospital of Southern Denmark, Odense, Denmark

**Keywords:** preeclampsia, disease severity, contact activation, kallikrein generation, cleaved H-kininogen

## Abstract

**Objective:**

The pathophysiology of preeclampsia is not fully understood. Disturbances in the contact system are associated with preeclampsia. Few studies have investigated the association between preeclampsia and alterations in the contact system in plasma. This study aims to elucidate whether this basic biological system is affected in preeclampsia using new methods focusing on the dynamic interactions and total capacity of the contact system in blood.

**Design:**

Cross-sectional study matching women with preeclampsia and controls without preeclampsia regarding age, pregestational body mass index, and gestational age at onset of the disease.

**Setting:**

Two Danish University hospitals.

**Sample:**

A cohort of 117 women with preeclampsia and 117 controls.

**Methods:**

The turnover and capacity of the contact system were determined with new methods. Paired *t*-test, Wilcoxon signed-pairs signed rank test, Mann-Whitney or Chi^2^-test were applied, as appropriate.

**Main Outcome Measurements:**

Kallikrein generation (peak kallikrein concentration and endogenous kallikrein potential), coagulation factor XII, prekallikrein, H-kininogen, cleaved H-kininogen, and complement C1 esterase inhibitor.

**Results:**

The endogenous kallikrein potential, peak kallikrein concentration, prekallikrein and cleaved H-kininogen were significantly lower in women with preeclampsia compared to the controls, *p* ≤ 0.005, whereas the concentration of coagulation factor XII, H-kininogen and complement C1 esterase inhibitor was not significantly different, *p* > 0.05.

**Conclusion:**

This study demonstrates significant reduction in kallikrein generating capacity, prekallikrein and cleaved H-kininogen indicating that the contact system is affected in preeclampsia suggesting a link to the pathophysiology of the disease.

## Introduction

Worldwide, preeclampsia (PE) is a major contributor to maternal morbidity and mortality and can cause severe perinatal complications such as prematurity and intrauterine growth retardation (IUGR). Approximately 5% of all pregnancies are affected by PE (1), which occurs only during pregnancy or early post-partum. The incidence of preterm PE (before 37 weeks of gestation) has remained unchanged during the last decade (2) despite innumerable attempts of predicting the disease and providing prophylactic care. Still, the pathophysiology of PE is only partly understood, and the root cause is not clear. However, placenta dysfunction or reduced maternal cardiovascular adaption or a combination of these conditions may contribute to the pathophysiology of PE.

It has been reported, that proteins of the contact system (CAS) regulate the placental function, and demonstrated that components of CAS are present in human placenta, and that the system may contribute to the placental function by regulating blood flow and transplacental transport of metabolites (3). These observations prompted us to speculate whether CAS in blood plays a role in the development of PE. The plasma proteins coagulation factor XII (FXII), prekallikrein (PK) and H-kininogen (HK) are the constituents of CAS. The activity of the system is regulated by protease inhibitors, of which C1-esterase inhibitor (C1-inh) is of particular importance. CAS is initiated by the interaction between FXII, PK, and an activating substance. The interaction determines the further propagation of CAS (4) which may lead to activation of coagulation, inflammation or fibrinolysis. By these actions, CAS may contribute to the maternal pro-thrombotic state, the fibrinolytic changes and the increased inflammatory response characterizing PE (5, 6).

Conversely, only few studies have investigated the association between PE and alterations in the activation and regulation of CAS in plasma despite the possibility that disturbance in CAS may be associated with early gestational loss (7, 8) and adverse pregnancy outcomes like preterm birth (9) and PE (10–12). Few prospective and cross-sectional studies demonstrate that the plasma levels of CAS proteins are affected during pregnancies complicated by PE (10, 13–18). However, most of these studies are more than 30 years old, and the complex interactions characterizing the initiation and propagation of CAS cannot be deduced by the methods used previously. We have developed new methods focusing on the dynamic interactions of the CAS proteins and the total capacity of CAS in blood (19–21), thereby giving us the opportunity comprehensively to study whether this basic biological system is associated with the pathophysiology of PE.

Thus, the aim of the present cross-sectional study is to compare the plasma concentration of CAS proteins and the turnover and capacity of CAS in pregnant women suffering from PE and matched healthy pregnant women, in particular concerning the inflammatory pathway of CAS.

## Materials and Methods

### Patient Cohort

The Preeclampsia and ContAct System study (PreCAS) is a matched cross-sectional trial conducted from January 2020 to October 2021. Patients were included at Departments of Obstetrics and Gynecology at University Hospital of Southern Denmark, Esbjerg and Odense. The hospitals take care of ~6,000 births annually.

PE is defined by a national guideline from 2018 (22) as a combination of hypertension and proteinuria after 20 weeks of gestation or hypertension accompanied by one of the following: hematological or neurological complications, liver dysfunction, renal failure, pulmonary edema or utero placental insufficiency. The National Guideline is generally in accordance with the International Society for the Study of Hypertension in pregnancy guideline from 2018. Severe PE was defined as blood pressure > 160/100 mmHg and/or severe subjective symptoms or severity of the definition criteria (22). Preterm PE is defined as delivery before 37 weeks of gestation (2). Fetal growth restriction (FGR) is defined as fetal weight below −15% of expected (22). Pregnant women above the age of 18 fulfilling the diagnostic criteria of PE were enrolled in the study either in the outpatient clinic or when admitted to the hospital. One-hundred and seventeen women were asked to participate and all accepted.

For each women with PE, a healthy pregnant woman was included. The control subjects were matched with cases with respect to gestational week (GA) (±1 week), age (±1 year) and pregestational body mass index (BMI) (±1 kg/m^2^).

During the period of inclusion, 117 women with PE were enrolled. The control subjects were recruited and included in the study in relation to an antenatal care session and 1,044 pregnant women consented to be possible controls. Data on the matching criteria was obtained in a secure database. Controls with the best possible match on all three criteria were contacted by telephone and invited to participate. Women suffering from PE in a former pregnancy, receiving cortisone in current pregnancy or developed PE were excluded as controls.

**Figure 1:**
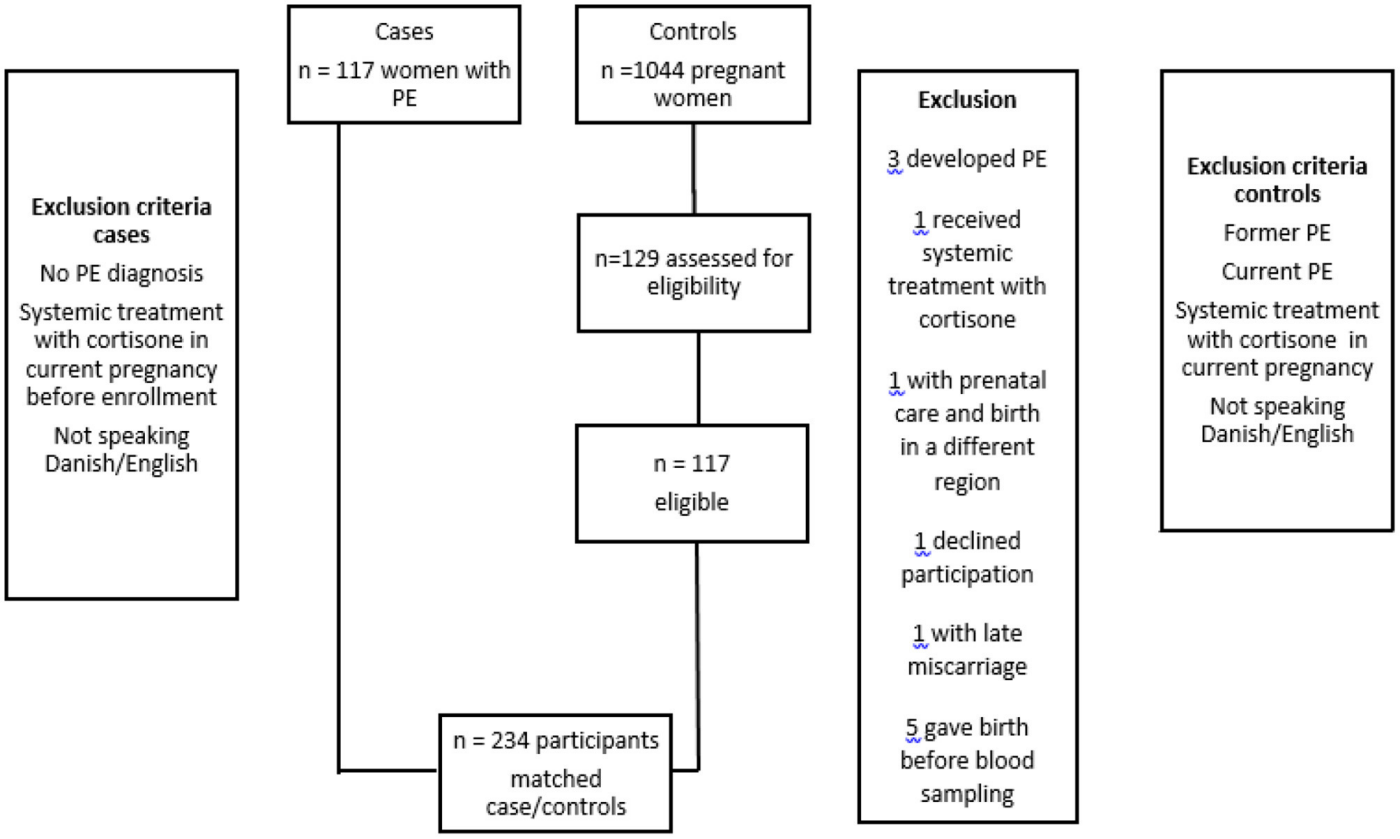
Patients with preeclampsia (PE) and controls enrolled in the PreCas-study. All invited PE-patients (117) accepted to participate. The control group consisted of 1,044 patients and 129 were assessed as eligible. Twelve controls were excluded from the study based on the exclusion criteria.

Of these 129 women were matched and invited to participate in the project. Twelve control subjects were excluded (Figure 1). Three women developed PE after blood sampling. One woman received systemic treatment with adrenal cortex hormone due to chronic inflammatory bowel disease. One woman had a severe medical condition and had prenatal care and delivery elsewhere. One woman suffered from late miscarriage and was not pregnant when contacted. Five women gave birth before blood sampling. One woman declined participation.

### Details of Ethics Approval

Informed written consent was obtained from the participants before inclusion. The study was conducted according to the Helsinki declaration including study approval by the Danish Data Protection Agency. Data from study participants are protected according to the Act on Processing of Personal Data. The Regional Ethics Committee in the Region of Southern Denmark approved the project (project ID S-20190142, 10 December 2019). The project was registered at www.clinicaltrials.gov as NCT04825145.

### Blood Collection and Handling

Blood samples were collected from the patients upon inclusion when they fulfilled the above mentioned criteria for being diagnosed with PE and from the controls. The collection and handling of blood samples followed the G41 guideline from Clinical and Laboratory Standards Institute (CLSI) (23) with specific focus on the recommendations for collection, transport and processing of blood specimens for testing plasma-based coagulation assays as detailed in the H21-A5 guideline from CLSI (24).

In brief, 16.8 ml blood was collected from an antecubital vein in four evacuated 2.7 ml tubes containing 0.105 mol/L sodium citrate (Vacutainer 9NC, Becton Dickinson, Plymouth, UK) and two evacuated 3 ml tubes containing 5.4 mg dipotassium-ethylene-diamine-tetra-acetate (EDTA) (Vacutainer K2E, Becton Dickinson). Platelet poor plasma was collected after centrifugation for 20 min at 2,000 × g. The citrate and EDTA-stabilized plasma samples, respectively, were subsequently stored at −80°C in tightly capped cryotubes (Sarstedt, VWR-Bie & Berntsen, Søborg, Denmark). Before analysis, the samples were thawed for 5 min at 37°C, kept at room temperature, and analyzed within 1 h.

### Laboratory Assays

Measures of kallikrein (PKa) generation, i.e., the lag time, time to peak, peak PKa concentration, and endogenous PKa potential (EKP) were recorded by an automated PKa generation assay as previously reported (19).

An in-house enzyme-linked immunosorbent assay (ELISA) that specifically recognizes cleaved HK (cHK), was used to measure the plasma concentration of cHK. Briefly, 96-well polystyrene flat bottom MicroWell™ MaxiSorp™ plates (Thermo Fisher Scientific, Lillerød, Denmark) were coated with 2 μg cHK mAb 19-20-8 per ml. The citrate plasma samples were analyzed at a 1:100 dilution and calibrated against kininogen depleted plasma (Affinity Biologicals, Ancaster, Ontario, Canada) spiked with 10 mg/ml purified cHK purchased from Enzyme Research Laboratories, Inc. South Bend, IN, USA. Biotinylated cHK mAb 19-31-18 (0.14 μg/ml) was used as detection antibody, and plates were visualized using HRP-conjugated streptavidin and ultra TMB ELISA substrate (Kementek, Taastrup, Denmark). The plates were read at 450 nm using a Tecan Sunrise ELISA reader (Tecan, Männedorf, Switzerland).

The protein concentration of HK was assessed by in-house prepared ELISA employing specific monoclonal antibodies. Briefly, 96-well polystyrene flat-button MicroWell MaxiSorp plates were coated with 1.0 μg Mab HK-6 per ml. Plasma samples were analyzed at a 1:4.000 dilution and a citrate plasma pool from 30 healthy volunteers was used as a calibrator. Biotinylated Mab HK 19-31-18 (0.14 μg/ml) was used as a detection antibody and plates were visualized using horseradish peroxidase-conjugated streptavidin and the TMB One-substrate (Kementek). The plates were read at 450 nm with 650 nm as reference using the Tecan Sunrise ELISA reader.

The plasma protein concentration of FXII and PK was determined with ELISA as described previously (20, 21).

The protein concentration of C1 inh was determined using N antiserum against human C1 inh, buffers, and reagents, employing the BN II analyser (all from Siemens Healthcare Diagnostics, Marburg, Germany).

### Statistics

The power calculation was based on the EKP, i.e., the total amount of PKa that can be generated in plasma after contact activation (19). The mean EKP determined in a reference population (*n* = 85) is 2,172 mIU/ml × min with a standard deviation of 515 mIU/ml × min. Aiming at a change in EKP of 10%, and using an alpha-value of 0.05 and a beta-value of 80% revealed that 2 × 90 women should be included in the study. The maximal dropout frequency was stipulated to 25% and consequently 2 × 113 women had to be included in the study.

Statistical calculations were performed by GraphPad Prism version 9.1.2 (GraphPad Software, San Diego, CA, USA). The distribution of the results were verified by a QQ plot and the Kolmogorov-Smirnov test. Paired *t*-test, Wilcoxon matched-pairs signed rank test, unpaired *t*-test, Mann-Whitney or Chi^2^-test were applied when appropriate. Results are presented as mean ± standard deviation or median and 25–75 percentile range, as appropriate. A *p*-value < 0.05 was considered statistical significant.

## Results

Women with PE were comparable with controls with respect to the matching criteria, maternal age, pregestational BMI and gestational age (Table 1).

**Table 1:** Matching criteria.

	**Women with preeclampsia** ***n* = 117**	**Controls** ***n* = 117**
Age (years)	29 (20–42)	29 (20–42)
Pregestational BMI (kg/m^2^)	27.5 (16.5–50.0)	27.4 (16.0–48.2)
Gestational age for PE (weeks + days)	37+3 (28+3–41+6)	37+3 (29+0–40+6)

*The results are presented as median (min-max)*.

*BMI, Body mass index; PE, preeclampsia*.

The characteristics of the study participants are shown in Table 2.

**Table 2:** Characteristics of the study participants.

	**Women with preeclampsia *n* = 117**	**Controls** ***n* = 117**	***p*-value**
Pregnancy (number)	2 (1–9)	2 (1–11)	0.15
Nullipara	56.4%	44.4%	0.07
Ethnicity	94.9% Caucasian	97.4% Caucasian	0.40
	3.4% Afro-Caribbean	0.9% Afro-Caribbean	
	1.7% East-Asian	1.7% East-Asian	
Smoking status	81.2% non-smokers	91.5% non-smokers	0.028
	12.8% former smokers	3.4% former smokers	
	6.0% current smokers	5.1% current smokers	
Current pregnancy singleton/multifetal	93.2% singleton	96.6% singleton	0.24
	6.8% gemelli	3.4% gemelli	
Blood pressure in early pregnancy (mmHg)	Systolic: 124 ± 12	Systolic: 117 ± 12	<0.0001
	Diastolic: 81 ± 10	Diastolic: 75 (48–96)	
Baby birth-weight (g)	2,992 ± 688	3,612 ± 549	<0.0001
Gestational age at delivery	38+2 (30+1–42+0)	40+3 (35+5–42+3)	<0.0001
Assisted reproductive treatment	12.0%	9.5%	0.54

*The results are presented as mean ± SD or median (min-max), as appropriate*.

No significant differences were seen in the number of nulliparous women in women with PE and controls (*p* = 0.07).

In early pregnancy the blood pressure was higher in the women developing PE, than in the control women (*p* < 0.0001). The weight of the newborn was lower (*p* < 0.0001) and the duration of pregnancy was shorter (*p* < 0.0001) in women with PE compared to their controls. No difference was seen in in the number of pregnant women who had received assisted reproductive treatment (*p* = 0.54).

Thirty-four of the women with PE 34 had severe disease and 29 had preterm PE. Fifteen of the women had both severe disease and preterm PE.

**Figure 2:**
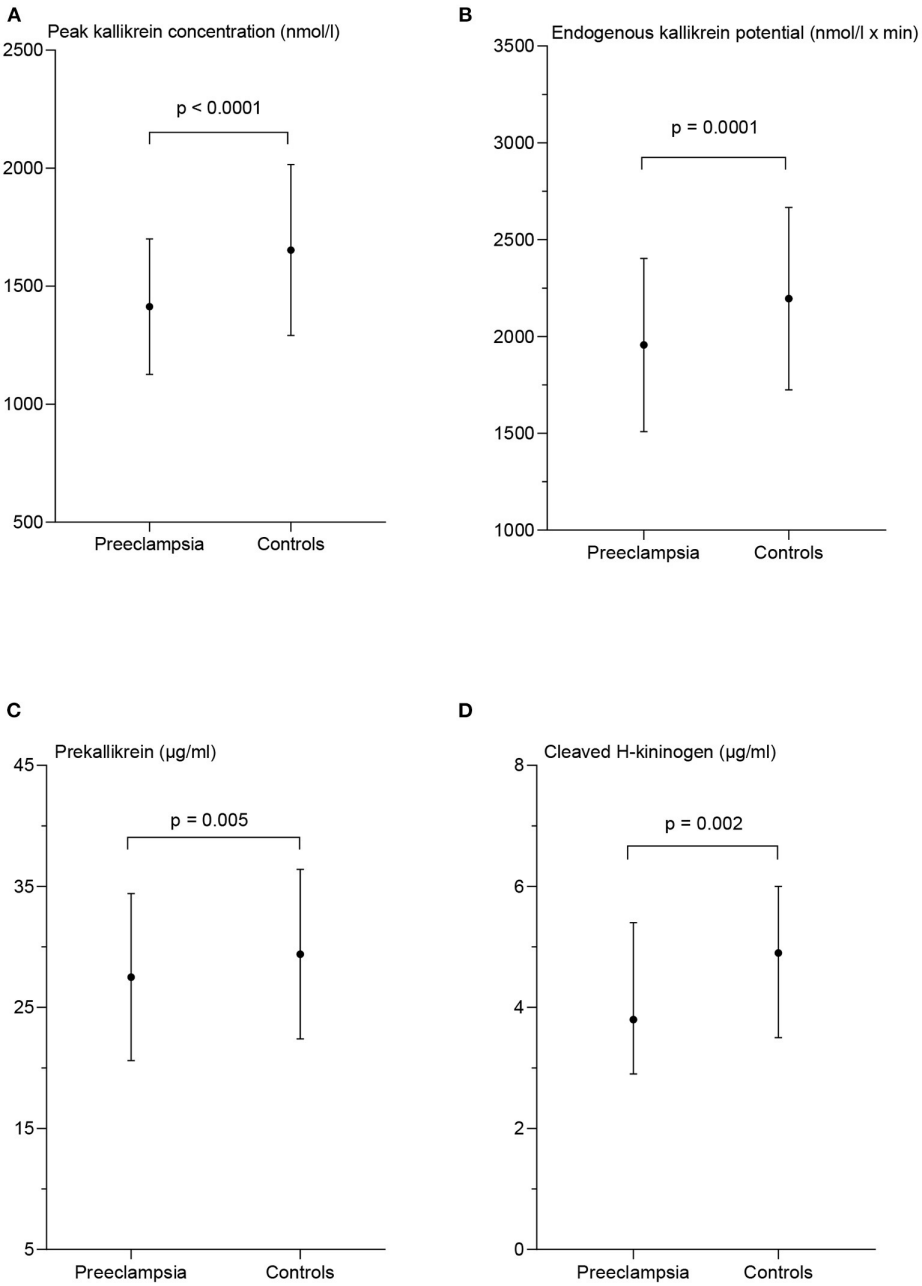
Measures of contact activation in patients with preeclampsia and matched controls. Peak kallikrein concentration **(A)**, endogenous kallikrein potential **(B)**, and prekallikrein concentration **(C)** are presented as mean ± standard deviation. The concentration of cleaved H-kininogen **(D)** is presented as median and interquartile range. *p*-values from the paired *t*-test **(A–C)** and the Wilcoxon matched-pairs signed rank test **(D)** between the two patient groups.

The peak PKa concentration was significantly lower in the PE-women compared with controls; 1,413 nmol/l ± 287 vs. 1,653 nmol/l ± 362, *p* < 0.0001. The EKP was significantly lower in the PE-women compared to the controls; 1,956 ± nmol/l × min ± 447 and 2,195 nmol/l × min ± 471, respectively, *p* = 0.0001 (Figure 2). In addition, the lag time and the time to peak of PKa generation were significantly longer in PE-women than in controls, *p* = 0.03 and *p* = 0.02, respectively (data not shown).

The women in the PE group had significantly lower PK concentration than the controls; 27.5 μg/ml ± 6.9 and 29.4 μg/ml ± 7.0 respectively, *p* = 0.005 and the plasma concentration of cHK was also significantly lower in the PE group with a median of 3.8 μg/ml (2.9–5.4) than the controls; 4.9 μg/ml (3.5–6.0), *p* = 0.002 (Figure 2).

**Figure 3:**
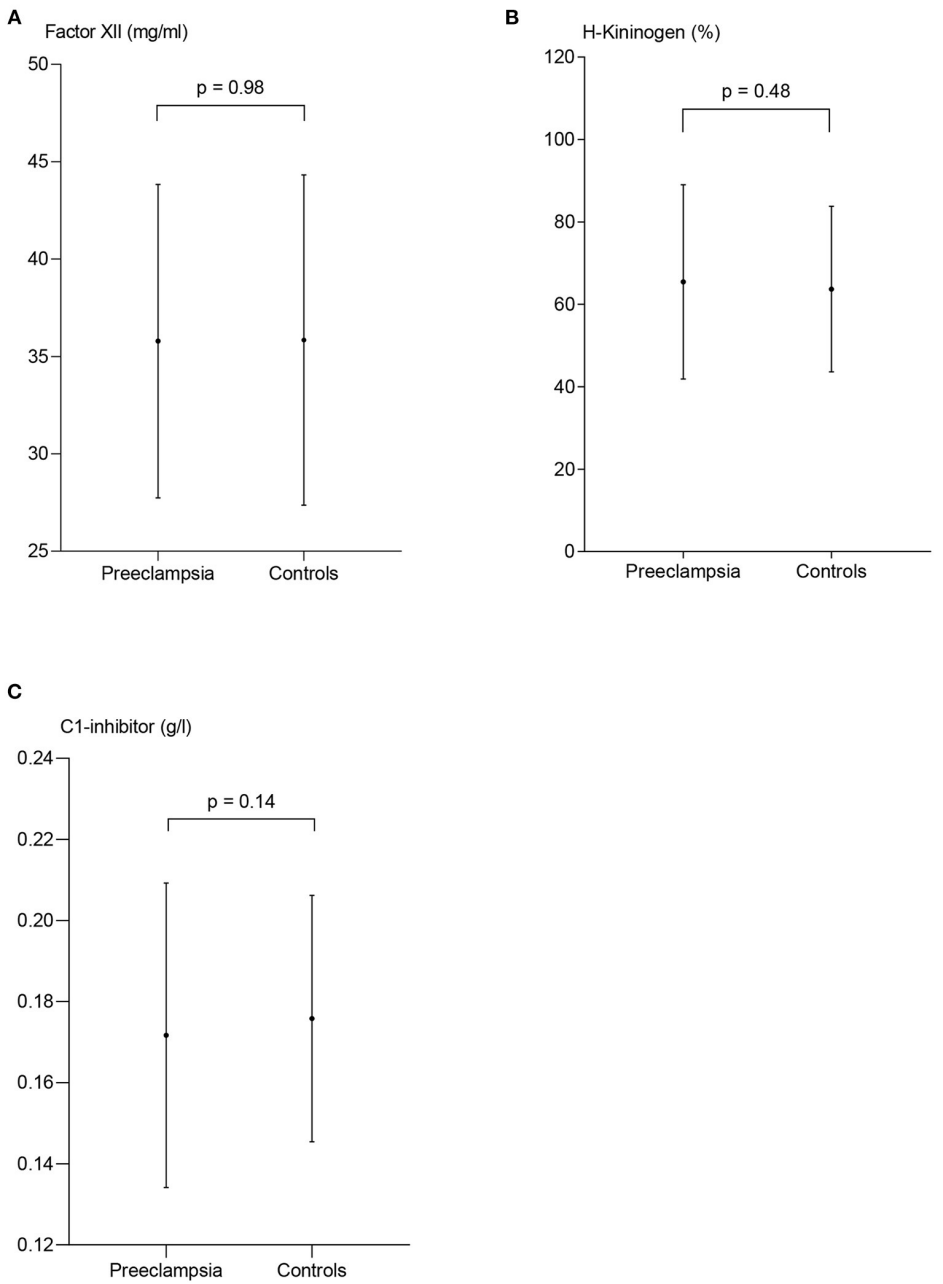
Measures of contact activation in patients with preeclampsia and controls. The plasma concentration of coagulation factor XII (FXII) **(A)** and H-kininogen **(B)** are presented as mean ± standard deviation. C1-esterase inhibitor **(C)** is presented as median and interquartile range, p-values from the paired t-test **(A,B)** and the Wilcoxon matched-pairs signed rank test **(C)** between the two patient groups.

The plasma concentrations of FXII, HK and C1-inh in women with PE were 35.8 mg/l ± 8.1, 65.5% ± 23.6, and 0.17 g/l (0.15–0.19), respectively, and not significantly different from control subjects where the corresponding concentrations were 35.8 mg/l ± 8.5, 63.7 ± 20.1, and 0.17 g/l (0.16–0.19), *p* = 0.98, *p* = 0.48, and *p* = 0.14, respectively (Figure 3).

**Figure 4:**
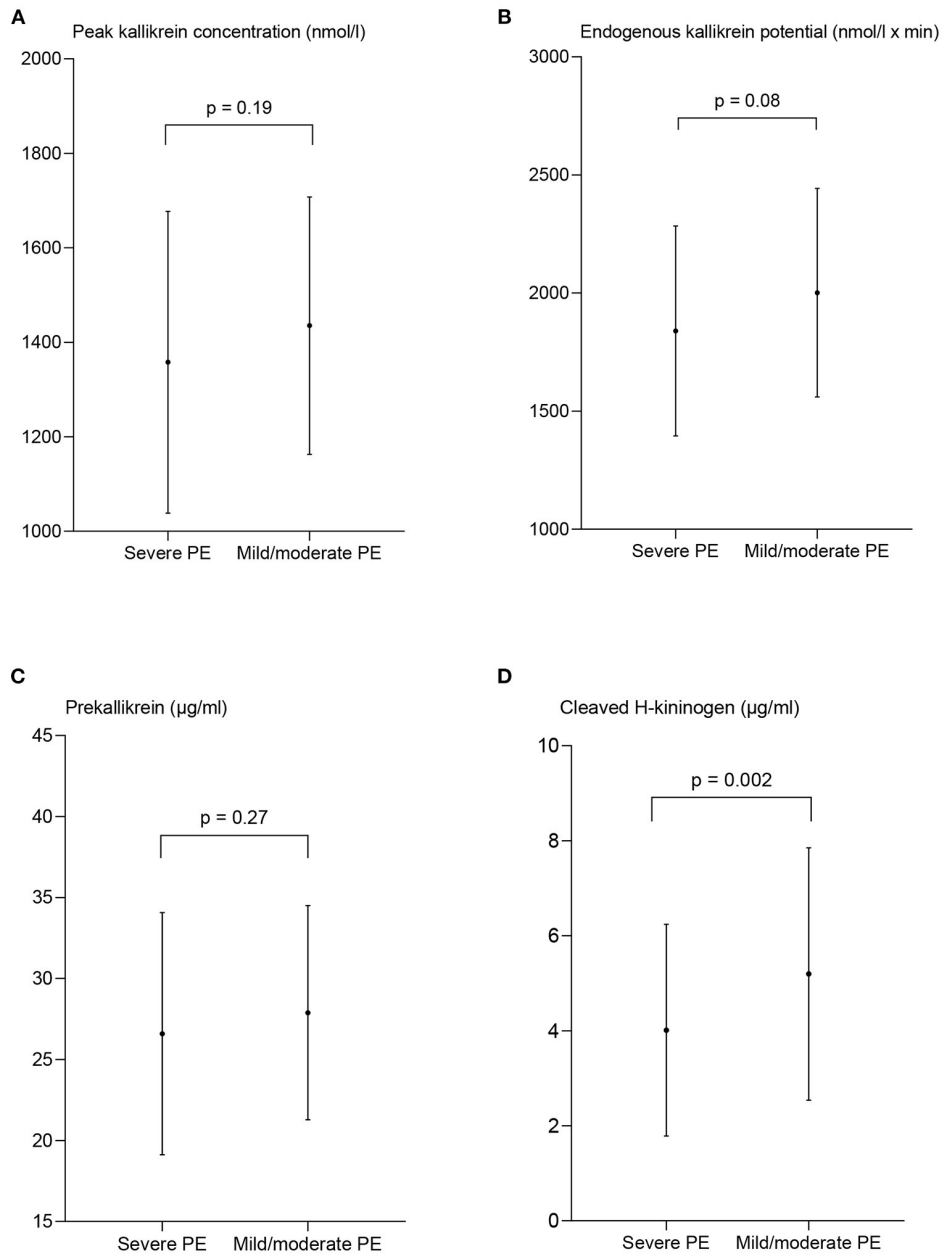
Measures of contact activation in women with severe and mild/moderate preeclampsia (PE). The peak kallikrein concentration **(A)**, endogenous kallikrein potential **(B)**, and prekallikrein concentration **(C)** are presented as mean ± standard deviation. The concentration of cleaved H-kininogen **(D)** is presented as median and interquartile range. *p*-values from the paired *t*-test **(A–C)** and the Wilcoxon matched-pairs signed rank test **(D)** between the two patient groups.

**Figure 5:**
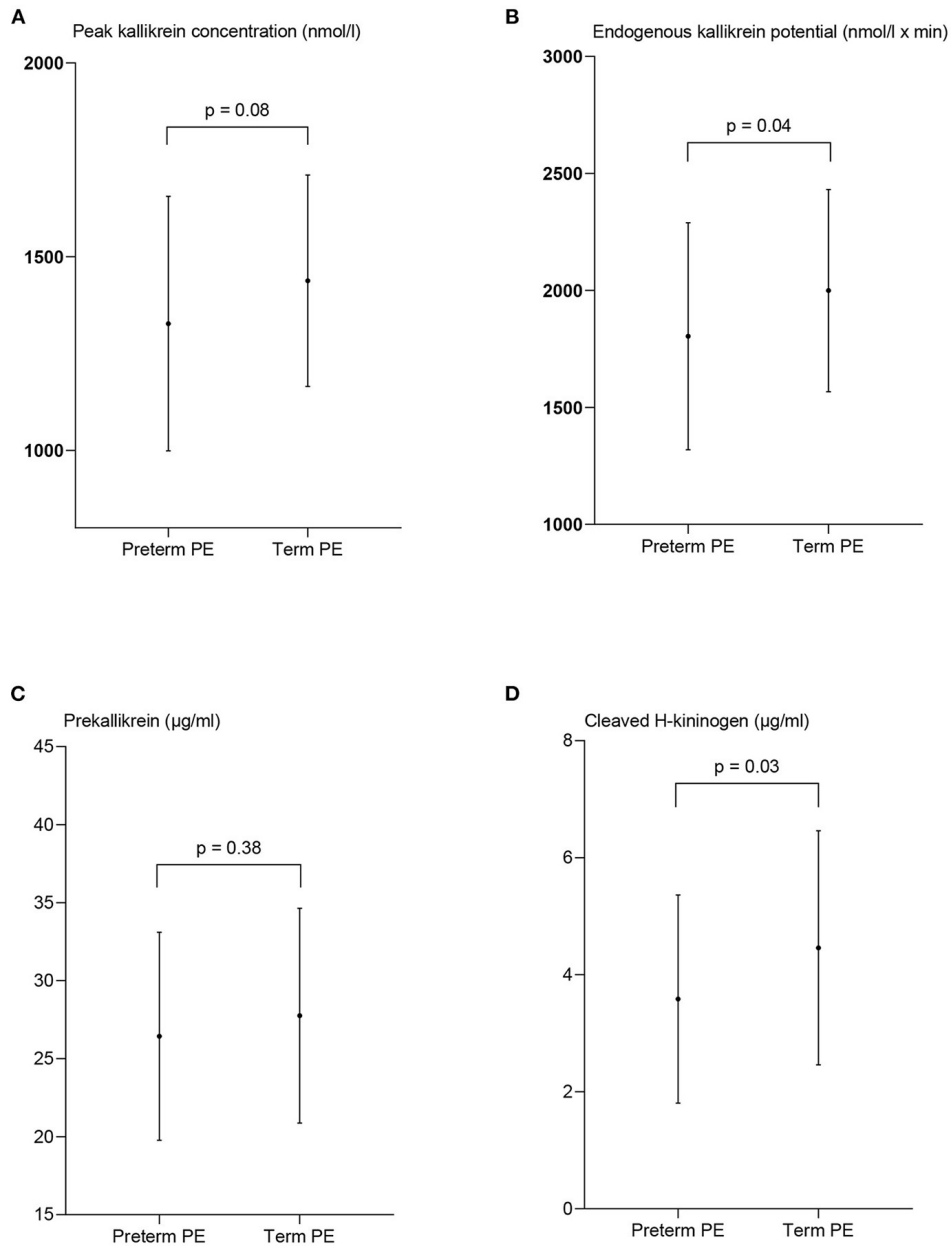
Measures of contact activation in women with preterm preeclampsia and term preeclampsia (PE). The peak kallikrein concentration **(A)**, endogenous kallikrein potential **(B)**, prekallikrein concentration **(C)**, and the concentration of cleaved H-kininogen **(D)** are presented as mean ± standard deviation. *p*-values from the paired *t*-test between the two patient groups.

Sub analyses of the PE group were performed, and peak PKa, EKP, the plasma concentration of PK, and cHK in women with severe and preterm PE are shown in Figures 4, 5.

The peak PKa concentration in women with severe PE, 1,358 nmol/l ± 320 did not deviate significantly from the women with mild/moderate PE, 1,435 nmol/l ± 272 (*p* = 0.19). Also EKP was comparable in women with severe PE, 1,840 nmol/l × min ± 445 and women with mild/moderate PE, 2,002 nmol/l × min ± 442, *p* = 0.08 (Figure 4).

The concentration of cHK was significantly reduced in the women with severe PE, 3.37 μg/ml (2.47–5.22) compared to the women with mild/moderate PE, 4.94 μg/ml (3.66–5.95), *p* = 0.002. The PK concentration was not significantly different among women with severe PE, 26.6 μg/ml ±7.5, and women with mild/moderate PE, 27.9 μg/ml ± 6.6, *p* = 0.27.

The peak PKa concentration was lower in women with preterm PE 1,327 nmol/l ± 329 than term PE, 1,438 nmol/l ± 273, although not significant (*p* = 0.08). EKP was significantly lower in women with preterm PE than in women with term PE, 1,804 nmol/l ± 485 vs. 1,999 ± 432, *p* = 0.04.

The concentration of cHK was significantly reduced in the women with preterm PE compared to the women with term PE (*p* = 0.03) with a concentration of 3.6 μg/ml ± 1.8 compared to 5.1 μg/ml ± 3.2, *p* = 0.03. The PK concentration was not significantly different between the groups; 26.4 μg/ml ± 6.7 in the preterm group and 27.8 μg/ml ± 6.9 in the term group, *p* = 0.38 (Figure 5).

Of the 117 women with PE included in the study, 22 had FGR. Sub analysis showed no significant difference in measures of CAS between women with PE and FGR and women with PE and without FGR.

## Discussion

### Main Findings

The present trial is the first matched cross-sectional study addressing the potential association between CAS and PE. Only few studies have investigated the association between PE and alterations in the activation and regulation of CAS in plasma (10, 13–18). Most studies are more than 30 years old, and it is not possible to reveal the complex protein interactions, including regulation and propagation of CAS with the methods used previously. Using a matched design and a more advanced analytic setup, we presently demonstrate that CAS is affected in pregnant women suffering from PE.

The key findings are that both the peak PKa concentration and the PKa generating capacity are reduced in women with PE compared to their controls, suggesting that CAS capacity is significantly depressed in PE patients. Accordingly, we observe that cHK is decreased in women with PE, and additionally decreased in women with severe disease or preterm PE.

### Strengths and Limitations

The strength of this study is the advanced analytical setup and the matched design. The PKa generating capacity and the concentration of CAS proteins might be dependent on the maternal and gestational age and the maternal BMI. Conversely, our design prevents the potential bias caused by these factors, and in addition the number of participants in our study are considerably higher than in former studies. On the other hand, the cross sectional design is a limitation. It would have been profitable to use a prospective study design to reveal the activation of CAS even before onset of PE, but this would require a much larger number of participants. Moreover, the observed effects on CAS in PE women could be related to the disease or a genuine condition in these women. This might contribute to the explanation of the increased risk of cardiovascular disease later in life in this group of patients (25). A follow up period could therefore be beneficial to shed light on the behavior of CAS beyond pregnancy.

We separated the PE patients into sub-groups with respect to severity of the disease and gestational age at delivery. The sizes of these groups were rather small, and the statistical power is not sufficient to allow firm conclusions. It has, however, been suggested that the pathophysiology of PE might be different in the groups regarding placental dysfunction and reduced maternal cardiovascular adaption to pregnancy (25, 26). Nonetheless, subgroup analysis regarding severity of PE and gestational age at delivery revealed trends in the key findings toward more extensive changes of CAS.

### Interpretation

PKa is formed through activation of PK and PKa cleaves HK thereby releasing bradykinin and the two-chain molecule cHK that is a specific marker for CAS activation. Accordingly, depressed cHK corresponds conceptually with the finding of reduced PKa generating capacity in the PE group as demonstrated with the PKa generation assay. Even further reduced PKa generating capacity was noticed in women with preterm PE.

The present study also demonstrates significantly lower plasma concentration of PK in patients with PE compared to the control group. This is in line with former studies (10, 14, 15). A small cross-sectional study (14) revealed lower levels of PK in women with PE than in late term pregnant women. Another cross-sectional study (10) compared normal pregnant women with women developing PE and subdivided this group with respect to the severity of the disease. A significantly lower PK was detected in women with PE, and the reduction in PK correlated to the severity of the disease. Therefore, a link between the level of PK and the pathophysiology of PE was proposed. One small cross-sectional study (15) showed no difference in the concentration of PK in women with PE compared to pregnant women without PE. The different result might be due to small sample size. These studies suggest that both the plasma concentration and the activity of PK are reduced in women with PE. The concentration of PK is of importance for PKa generation thereby helping to explain the observed depression in CAS capacity in pregnant women with PE compared to healthy pregnant women.

In contrast to a previous study (13), reporting increased levels of FXII in women with PE, we observe no significant difference in the plasma concentration of FXII between our two groups. However, in the previous study, the women with PE were not comparable to control subjects regarding age, BMI, and GA for PE as the patients included in our study. Moreover, the study employed a functional method for determination of FXII, whereas in our study the protein concentration of FXII was determined.

The potential relation between PE and HK has previously been addressed in only one small study (16) using a semi quantitative immunological method for detection of HK. Reduced levels of HK were observed in women with PE complicated with babies who were small for gestational age. In contrast, we quantified the plasma protein concentration of HK and observed no difference in the concentration of native HK between women with PE and controls.

The plasma concentration of C1-inh was not different in the two groups. Similarly, two small cross-sectional studies (17, 18) have investigated C1-inh in women with PE. Halbmayer et al. revealed no difference in PE women compared to pregnant women without PE, whereas Hsieh et al. found a decrease in women with PE. Both studies included only few participants, and the method used for determination of the C1-inh, was different than the method employed in the present study.

The reason for a depressed CAS capacity in women with PE is unclear. It could be speculated whether women with a genuine low CAS capacity have an increased risk of PE. In order to answer this question sufficiently there is a need for a large prospective study. Another explanation could be a consumption of CAS proteins in early pregnancy in the individuals later suffering from PE. Bryant and Shariat-Madar (27) considered the theory of consumption. Their study described a possible activation of CAS due to reduced levels of total kininogen (high- and low molecular weight HK) in the placentas of PE women (28), indicating consumption of the proteins. Interestingly, and in this context, it has been reported that women developing PE produce misfolded proteins (29, 30) and that misfolded proteins *per se* have the capability to activate CAS (31). A third tentative explanation could be that an affected liver function could lower the concentration of CAS proteins in plasma. However, among the CAS proteins, only the concentration of PK was reduced and lower PK levels were not observed in patients with PE complicated by liver affection (ALAT > 70 U/l).

The reduced capacity of CAS reported in the present study may reduce the fibrinolytic potential in women with PE compared to controls and by this action contribute to the increased thrombotic risk observed in women suffering from PE. The potential association between CAS and the fibrinolytic system will be addressed in future studies.

### Conclusion

The current study addresses changes in CAS proteins in women with PE compared to matched pregnant women without PE. The study demonstrates that the peak PKa concentration, the PKa generating capacity, and the concentration of cHK are significantly reduced in women with PE. The results suggest that CAS is affected in PE suggesting a contribution to the pathophysiology of the disease.

## Data Availability Statement

The raw data supporting the conclusions of this article will be made available by the authors, without undue reservation.

## Ethics Statement

The studies involving human participants were reviewed and approved by the Regional Ethics Committee in the Region of Southern Denmark (project ID S-20190142, 10 December 2019). The patients/participants provided their written informed consent to participate in this study.

## Author Contributions

JBG, JJS, ACG, JSJ, and YP designed and directed the research. ACG collected and managed data in collaboration with BBD. ACG conducted the statistical analyses. ACG and JJS wrote the initial draft. JJS, JBG, STDP, and YP developed methods for the study. All authors contributed to the article and approved the submitted version.

## Funding

This study was supported by Lida and Oskar Nielsens Foundation, Esbjerg Foundation, Gangsted Foundation, the Research Foundation of Southern Denmark, and Et Sundere Syddanmark.

## Conflict of Interest

The authors declare that the research was conducted in the absence of any commercial or financial relationships that could be construed as a potential conflict of interest.

## Publisher's Note

All claims expressed in this article are solely those of the authors and do not necessarily represent those of their affiliated organizations, or those of the publisher, the editors and the reviewers. Any product that may be evaluated in this article, or claim that may be made by its manufacturer, is not guaranteed or endorsed by the publisher.

## Abbreviations

BMI: Body mass index
CAS: contact activation system
cHK: cleaved H-kininogen
CLSI: Clinical and Laboratory Standards Institute
C1-inh: complement 1-esterase inhibitor
EDTA: dipotassium-ethylene-diamine-tetra-acetate
EKP: endogenous kallikrein potential
ELISA: enzyme-linked immunosorbent assay
FGR: fetal growth restriction
FXII: coagulation factor XII
GA: gestational age
HK: H-kininogen
HRP: Horseradish Peroxidase
IUGR: intrauterine growth retardation
Mab: monoclonal antibody
N: antiserum
PE: preeclampsia
Pka: kallikrein
PK: prekallikrein
PreCAS: The Preeclampsia and ContAct System study.
